# Quantification of Bacterial and Drug-Resistant DNA Using dPCR in a Pediatric Patient with CVC-Related Bloodstream Infection

**DOI:** 10.3390/idr17050130

**Published:** 2025-10-16

**Authors:** Masato Kojima, Hiroki Kitagawa, Kayoko Tadera, Ryo Touge, Sho Kurihara, Mari Tanaka, Maiko Shimomura, Isamu Saeki, Hiroki Ohge

**Affiliations:** 1 Department of Pediatric Surgery, Hiroshima University Hospital, Hiroshima 734-8551, Japan; 2 Department of Infectious Diseases, Hiroshima University Hospital, Hiroshima 734-8551, Japan; 3 Section of Clinical Laboratory, Division of Clinical Support, Hiroshima University Hospital, Hiroshima 734-8551, Japan; 4 Division of Laboratory Medicine, Hiroshima University Hospital, Hiroshima 734-8551, Japan; 5 Department of Pediatrics, Hiroshima University Hospital, Hiroshima 734-8551, Japan

**Keywords:** catheter-related bloodstream infection, pediatrics, digital PCR

## Abstract

**Background**: Digital polymerase chain reaction (dPCR) is a highly sensitive molecular method that allows rapid detection of bacterial DNA and resistance genes, requiring only a small blood volume. Although not a new technology, its application in pediatric patients with suspected catheter-related bloodstream infection (CRBSI) remains limited. **Case presentation**: A 16-year-old female, diagnosed with recurrent acute myelogenous leukemia, received re-induction chemotherapy through a peripherally inserted central venous catheter (PICC). The patient developed a fever, and the blood culture (BC) drawn from the PICC was positive for methicillin-resistant *S. epidermidis*, leading to suspicion of CRBSI. Several antibiotics were used, and the PICC was replaced. Eventually, the fever subsided, and the BC was negative after PICC removal. The levels of *S. epidermidis*-specific DNA sequences and *mecA* genes were correlated with the results of the BC and clinical course. Turnaround time was significantly shorter in dPCR (3.5 h) than in the BC (14–21 h); dPCR was performed using only 400 µL of blood. **Conclusions**: This case highlights the potential of dPCR as a complementary tool to conventional BCs in the management of pediatric CRBSI. dPCR may support rapid decision-making and monitoring of the treatment response, particularly when sample volumes are limited.

## 1. Introduction

Central venous catheters (CVCs) are essential in the management of pediatric patients, including low-birth-weight neonates, infants with short-gut syndrome, and children with cancer or chronic diseases [1]. However, CVCs are frequently associated with bloodstream infections (BSIs), which may require catheter removal and can even result in mortality [1]. The management of catheter-related BSIs (CRBSIs) remains challenging due to several factors, such as the rising prevalence of multidrug-resistant organisms, the choice of appropriate antimicrobial therapy, treatment duration, and the decision of whether to retain the catheter in situ [1]. Because CVC insertion in pediatric patients requires general anesthesia or sedation, effective antimicrobial therapy is especially important to achieve catheter salvage. Blood culture (BC) remains the gold standard for the diagnosis and management of BSIs [1,2], but it is limited by low sensitivity and long turnaround times [2]. Digital polymerase chain reaction (dPCR), a third-generation PCR technique, was developed in the 1990s and introduced into clinical practice more recently. dPCR is based on partitioning the DNA sample into thousands of microdroplets, each serving as an independent PCR reaction chamber, which allows absolute quantification of target DNA molecules. Compared with real-time quantitative PCR (RT-qPCR) and next-generation sequencing, dPCR offers higher sensitivity, shorter turnaround times, and lower costs. Quantitative measurement of bacterial DNA using dPCR has been shown to correlate with sepsis severity, disease progression, and treatment response [3]. Although dPCR itself is not a new technology, its use in pediatric cases of suspected CRBSI remains limited. Importantly, dPCR requires only a small volume of blood and can provide rapid information on both causative pathogens and resistance genes, which may be particularly advantageous in children. In this case report, we evaluated the utility of dPCR for the quantitative detection of bacterial and drug-resistance DNA in managing a pediatric patient with suspected CRBSI.

## 2. Presentation of Case

A 16-year-old girl underwent a bone marrow transplant for acute myelogenous leukemia (AML) with PICALM::MLLT10 and experienced a relapse five months after transplantation.

### 2.1. Materials and Methods 

#### 2.1.1. Blood Culture and Pathogen Identification 

One BC specimen of venous blood was collected from the peripherally inserted central venous catheter (PICC), according to routine clinical procedures. Whole blood (4–10 mL) was inoculated into a BC bottle (BacT/ALERT PF Plus bottle, bioMérieux, Marcy l’Étoile, France) and incubated at 37 °C in a BacT/ALERT^®^ Virtuo System (Version 3.02.00.2142, bioMérieux, Marcy l’Étoile, France). Once the system reported a positive signal, Gram staining was performed, followed by subculturing, as previously described [4]. Isolates from blood cultures were identified via matrix-assisted laser desorption/ionization time-of-flight mass spectrometry (MALDI-TOF MS), using a MALDI Biotyper Sirius system (Version 4.1.100, Bruker Daltonik GmbH, Bremen, Germany), as described previously [4]. The minimum inhibitory concentration (MIC) of antimicrobial agents was determined using the broth microdilution method, with IA40 MIC-i (Device number 2.0.0.0, Eiken Chemical Co., Ltd., Tokyo, Japan) with Dry Plates Eiken (Eiken Chemical Co., Ltd., Tokyo, Japan). This was interpreted according to the Clinical and Laboratory Standards Institute recommendations [5].

#### 2.1.2. Whole Blood DNA Extraction

Whole blood samples (1–1.5 mL), collected for blood tests in ethylenediaminetetraacetate anticoagulant tubes, were used for dPCR detection. DNA was extracted on the same day or the day after whole blood samples were collected. Precisely purified Achromopeptidase^®^ (FUJIFILM Wako Pure Chemical Corporation, Osaka, Japan) was added to the collected blood, to a final concentration of 8000 units/mL, and the suspension was further incubated for 120 min at 37 °C. Afterward, DNA was extracted from 400 µL of the blood sample treated with Achromopeptidase^®^ using the automatic extraction system magLEAD^®^ 6gC with magLEAD^®^ Dx SV reagent (Precision System Science Co., Ltd., Chiba, Japan) following the manufacturer’s instructions. The final eluate (50 µL) was stored at −80 °C until use.

#### 2.1.3. dPCR Testing

The primers and probes (Integrated DNA Technologies, Coralville, IA, USA) used to detect methicillin-resistant *Staphylococcus epidermidis* were as follows [6,7]:

*Staphylococcus epidermidis*

Forward: 5’-ATCAAAAAGTTGGCGAACCTTTTCA-3,’ 

Reverse: 5’-CAAAAGAGCGTGGAGAAAAGTATC-3,’ 

Probe: 5’-HEX-CCATTTGCATAGTCTGATTGCTCAAAGTCT-BHQ1-3’).

*mecA* gene

Forward: 5’-GCTCAAATTTCAAACAAAAATTTAGATAATG-3,’ 

Reverse: 5’-TGAAAGGATCTGTACTGGGTTAATCAGT-3,’ 

Probe: 5’-FAM-AGCTGATTCAGGTTACGGACAAGGTGA-BHQ1-3’).

All assays were performed using a QX100 Droplet Digital PCR system Bio-Rad Laboratories, Inc., Pleasanton, CA, USA), according to the manufacturer’s instructions. Each reaction contained 5 µL of template DNA, 17 µL of master mix containing ddPCR Supermix for probes (no dUPT), 0.9 µL each of the forward and reverse primers (10 µM), and 0.25 µL of the probe (10 µM). Droplets were generated using an automated droplet generator (Bio-Rad Laboratories, Inc., Pleasanton, CA, USA). PCR reactions were then performed using the following protocol: 95 °C for 10 mins, 40 cycles at 94 °C for 30 s, and 57 °C for 1 min, and a final cycle at 98 °C for 10 mins. 

The number of droplets containing target DNA molecules was determined using a QX100 droplet reader and Bio-Rad QuantaSoft software (Version 1.3.2.0, Bio-Rad Laboratories, Inc., Pleasanton, CA, USA). The presence of at least three copies per test was considered ddPCR-positive. The highest number of positive droplets in triplicate was used as the number of target molecules in the whole blood samples and is shown as copies/mL. The turnaround time for blood culture in our case was 14–21 h, whereas dPCR required only approximately 3 hours and 32 minutes from DNA extraction to the determination of the copy number of the target genes.

### 2.2. Clinical Course and Results of BC and dPCR

**Figure 1:**
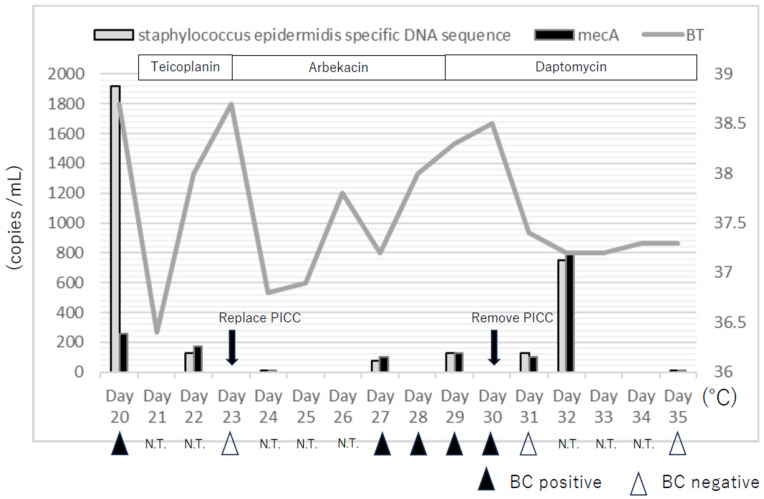
Clinical course, blood culture (BC) results, and digital polymerase chain reaction (dPCR). BT; body temperature BC; blood culture PICC; peripherally inserted central venous catheter N.T.; non-tested.

The clinical course and the results of BC and dPCR are shown in Figure 1. A PICC line was used for re-induction chemotherapy. The patient showed pancytopenia by re-induction chemotherapy and blood was taken from the PICC. Moreover, suppurative thrombophlebitis was suspected due to an induration at the insertion site of PICC. The patient developed fever on hospital day 20, with positive BC results from the PICC. Despite initial treatment with teicoplanin and subsequent changes in antibiotics, the patient experienced recurrent fever until the PICC was finally removed on day 30, after which the condition gradually improved. The detailed daily timeline of clinical course, BC, and dPCR results is presented in Figure 1.

**Figure 2:**
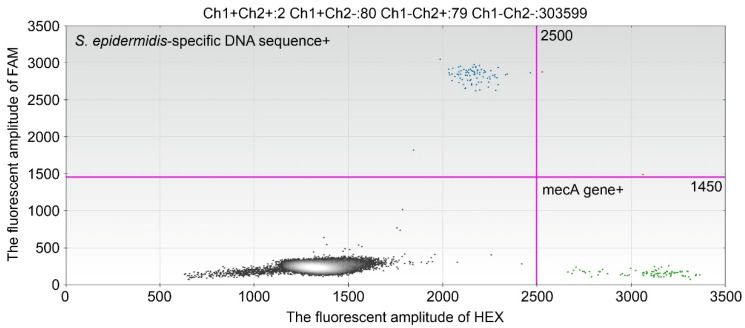
A typical dPCR result. The dPCR result on day 32 produced a *S. epidermidis*-specific DNA sequence of 750 copies/mL and *mecA* of 800 copies/mL.

On hospital day 20, the patient presented with a fever. BC of venous blood taken from a peripherally inserted central venous catheter (PICC) was positive (time to positivity (TTP) = 14 h) for methicillin-resistant *S. epidermidis*, and catheter-related bloodstream infection (CRBSI) was suspected. On the same day, 1920 copies/mL of *S. epidermidis*-specific DNA sequence and 260 copies/mL of the *mecA* gene were detected by dPCR in venous blood collected from the PICC. On day 21, the administration of teicoplanin (15 mg/kg/day) was started. On day 22, the copies of *S. epidermidis*-specific DNA sequence and *mecA* gene decreased and 125 copies/mL of *S. epidermidis*-specific DNA sequence and 175 copies/mL of the *mecA* gene were detected in venous blood collected from the PICC. On day 23, the BC of the venous blood taken from the PICC was negative, and teicoplanin was discontinued and arbekacin (5 mg/kg/day) initiated due to drug rush. In addition, we replaced the PICC on the same site. On day 24, *S. epidermidis*-specific DNA sequence and *mecA* genes were not detected in venous blood collected from the PICC. Although the fever gradually decreased, it increased from day 28. On day 29, the administration of daptomycin (6 mg/kg/day) instead of arbekacin was started. On day 30, the replaced PICC was removed because the BC of the venous blood taken from the PICC remained positive (day 27; TTP = 15 h, day 28; TTP = 18 h, day 29; TTP = 19 h, day 30; TTP = 21 h). In addition, the *S. epidermidis*-specific DNA sequence and *mecA* genes were also detected in the venous blood taken from the PICC (day 27; *S. epidermidis*-specific DNA sequence = 75 copies/mL, *mecA* gene = 100 copies/mL, day 29; *S. epidermidis*-specific DNA sequence = 125 copies/mL, *mecA* gene = 125 copies/mL). Thereafter, the fever gradually declined, and a negative BC of peripheral venous blood was confirmed on days 31 and 35. *S. epidermidis*-specific DNA sequence and *mecA* genes were detected in the peripheral venous blood on days 31 and 32 (day 31; *S epidermidis*-specific DNA sequence = 125 copies/mL, *mecA* gene = 100 copies/mL, day 32; *S. epidermidis*-specific DNA sequence = 750 copies/mL, *mecA* = 800 copies/mL). (Figure 2) However, a negative *S. epidermidis*-specific DNA sequence and *mecA* genes in peripheral venous blood was confirmed on day 35. We continued daptomycin administration until day 37, with a negative BC of peripheral venous blood.

## 3. Discussion

In this study, synchronous results between dPCR and BC were limited; however, when BC was positive, dPCR was also positive, and dPCR remained positive even when BC was negative. In particular, on the day after dPCR was positive, BT increased, and the BSI was more severe. The higher sensitivity of dPCR than BC was consistent with a previous study [8]. However, it should also be emphasized that while blood culture requires viable bacteria to yield a positive result, dPCR can detect bacterial DNA fragments even after the organisms have been killed by antimicrobial therapy. This explains why dPCR may remain positive for longer periods even when blood culture has turned negative. The rapid increase in targeted DNA by dPCR on day 32 may represent transient release of DNA from non-viable bacteria into the bloodstream caused by thrombolysis of suppurative thrombophlebitis after PICC removal. We also emphasize that dPCR should not be regarded as a reliable tool for confirming bacterial eradication. Because dPCR can detect DNA fragments from non-viable bacteria or resistance genes that are not necessarily linked to active infection, its results may persist even when viable organisms have been cleared. From a clinical perspective, this means that dPCR cannot determine eradication, and over-interpretation could potentially lead to unnecessary or inappropriate antimicrobial treatment. Therefore, dPCR results must always be interpreted in conjunction with clinical findings and blood culture data, and should be considered complementary rather than definitive.

Because dPCR can detect minute amounts of DNA, false-positive results due to contamination or residual DNA are another concern. In this case, *S. epidermidis* was continuously detected using BC and dPCR, while other organisms were not detected during the course of the disease. Although there is little possibility of contamination, *S. epidermidis* exists ubiquitously on the skin, and a cautious interpretation of results is needed. Strict adherence to aseptic sampling, inclusion of negative controls, and the use of internal quality controls are necessary to minimize false-positive results. Re-testing of borderline positive samples can also help confirm reproducibility.

The turnaround time was significantly shorter with dPCR than with BC, which can facilitate rapid decision-making in the management of CRBSIs. This rapidity is particularly advantageous during the first febrile episode or early in suspected infection, when viable bacteria are more likely to be present and timely treatment decisions are critical. In this setting, dPCR may potentially contribute to therapeutic decisions in several ways: rapid detection of resistance genes, such as *mecA*, could support the early choice of an appropriate antimicrobial agent; serial monitoring of bacterial DNA copy numbers might provide information on the duration of therapy, although such results must be interpreted with caution in accordance with existing guidelines for hospital-acquired infections, since DNA from non-viable or non-causative bacteria may persist; and persistently positive dPCR results despite adequate antimicrobial therapy might provide additional evidence to support catheter removal, particularly when consistent with clinical findings and blood culture data. However, these applications remain hypothetical and should be considered limitations of the present case report, as current evidence is insufficient to draw definitive conclusions. Nevertheless, the development of multiplex panels with species-specific primers and probes could further enhance the clinical utility of dPCR by enabling differentiation among clinically relevant organisms, thereby facilitating more targeted empirical therapy and potentially helping to limit the emergence of antibiotic resistance. In the present study, we employed primers and probes designed on the basis of BC results; however, preparing panels for other clinically important organisms, such as *Staphylococcus aureus*, in advance would allow the application of dPCR independent of BC. Indeed, Liu et al. recently demonstrated the clinical utility of preset primer/probe panels for several viruses using multiplex digital PCR in the rapid diagnosis of suspected pediatric bloodstream infections [9].

A definitive diagnosis of CRBSI is obtained mainly by comparative culture and differential time-to-positivity [1]. CRBSI is diagnosed when BC via the CVC, becomes positive at least 2 h before BC, via the peripheral blood [1]. In pediatrics, collecting adequate blood for BC is sometimes difficult. In our case, paired blood cultures were not obtained, and therefore the diagnosis of CRBSI could not be confirmed by strict laboratory criteria. Thus, our patient should be regarded as having a suspected rather than a laboratory-confirmed CRBSI, which represents an important limitation of this report. Nevertheless, dPCR required only a minimum of 400 µL of blood, and provided quantitative information that contributed to clinical management despite this limitation. Previous larger studies have already demonstrated the potential utility of dPCR in bloodstream infections [10,11,12]. Our report adds a pediatric case perspective, but further multicenter studies are required to validate the role of dPCR compared with conventional BC in clinical practice.

In this case, the patient received chemotherapy and developed neutropenia and body temperature was the leading indicator of the effectiveness of the antimicrobial therapy, apart from the BC results. In patients with neutropenia, quantitatively measuring bacterial DNA using dPCR can help to monitor the severity of BSI, and the effectiveness of antimicrobial therapy. In this case, the copy numbers of the *S. epidermidis* specific sequence and *mecA* gene in the blood were high even after the PICC was removed. Default DNA clearance is associated with treatment failure, and these results will help to determine the duration of antimicrobial therapy [3].

Myeloablative chemotherapy in pediatric patients with malignancies frequently causes febrile neutropenia, which is managed using empirical broad-spectrum antibiotics. The use of empirical broad-spectrum antibiotics increases the number of multidrug-resistant organisms. Infections by multidrug-resistant organisms are associated with high mortality in immunocompromised pediatric patients with malignancies. In this case, we detected the methicillin-resistance gene *mecA* in the blood using dPCR. This approach could also be applied to other drug-resistant genes, such as the carbapenem-resistance gene *blaKPC* and the vancomycin-resistance gene *vanA* [13]. However, it is important to note that the mere presence of such genes does not necessarily indicate phenotypic resistance, as expression and resistance may be influenced by insertion sequences, mutations, or regulatory mechanisms. Therefore, dPCR findings of resistance genes should be interpreted cautiously and always in parallel with conventional phenotypic susceptibility testing, such as MIC determination by culture, as well as the patient’s clinical course. This combined approach would help ensure that the detection of resistance determinants by dPCR contributes meaningfully to appropriate antimicrobial decision-making in pediatric malignancies.

Although dPCR offers high sensitivity and rapid turnaround time, there are several limitations. First, dPCR is unable to provide information on antimicrobial susceptibility beyond the specific resistance genes targeted by primers. Therefore, in cases involving unexpected or novel resistance mechanisms, culture-based methods remain indispensable. Second, dPCR is not suitable when broad-range pathogen identification is required, such as in polymicrobial bloodstream infections or in patients with atypical clinical presentations. Unlike dPCR, blood culture currently allows comprehensive and unbiased detection of a wide range of organisms, including unexpected pathogens. This represents a major limitation of dPCR at present. However, with the development of multiplex primer/probe panels targeting multiple clinically relevant organisms and advances in broad-range digital PCR approaches, dPCR could eventually overcome this limitation and potentially serve as an alternative to blood culture in the future, offering rapid and highly sensitive detection across a broader spectrum of pathogens.

Another important limitation is cost. dPCR instruments and reagents remain relatively expensive, and their implementation in routine hospital practice may be challenging, particularly in low- and middle-income countries. Moreover, specialized technical expertise is required for assay setup and data interpretation. Large-scale health-economic evaluations are warranted to assess whether the rapid turnaround time and reduced empiric antibiotic use can offset these costs in routine pediatric care.

Finally, we emphasize that dPCR should be considered a complementary diagnostic tool rather than a replacement for blood culture. While dPCR can rapidly provide information on pathogen DNA and resistance genes, blood culture remains essential for isolating organisms, performing antimicrobial susceptibility testing, and supporting epidemiological surveillance. Future studies using animal models, such as *S. epidermidis* bacteremia in immunodeficient mice, also could help establish the translational utility of dPCR in bloodstream infections.

In conclusion, dPCR enables quantitative measurement of bacterial DNA and may facilitate rapid clinical decision-making in the management of CRBSI, as well as real-time monitoring of the therapeutic response. Its applicability in pediatrics is enhanced by the low blood volume requirement compared with BCs. Importantly, although dPCR can rapidly detect causative pathogens and resistance genes, it should be interpreted alongside conventional blood cultures, which remain indispensable for definitive diagnosis and antimicrobial susceptibility testing.

## Data Availability

The datasets generated and/or analyzed during the current study are not publicly available but are available from the corresponding author on reasonable request.
